# Global Variability in Deep Brain Stimulation Practices for Parkinson’s Disease

**DOI:** 10.3389/fnhum.2021.667035

**Published:** 2021-03-31

**Authors:** Abhimanyu Mahajan, Ankur Butala, Michael S. Okun, Zoltan Mari, Kelly A. Mills

**Affiliations:** ^1^Rush Parkinson’s Disease and Movement Disorders Program, Chicago, IL, United States; ^2^Departments of Psychiatry and Neurology (GMP), Johns Hopkins University School of Medicine, Baltimore, MD, United States; ^3^Norman Fixel Institute for Neurological Diseases, Department of Neurology, University of Florida, Gainesville, FL, United States; ^4^Cleveland Clinic Luo Ruvo Center for Brain Health, Las Vegas, NV, United States; ^5^Department of Neurology, Johns Hopkins University School of Medicine, Baltimore, MD, United States

**Keywords:** DBS (deep brain stimulation), Parkinson’s disease, intra-operative, practices, international

## Abstract

**Introduction:**

Deep brain stimulation (DBS) has become a standard treatment option for select patients with Parkinson’s disease (PD). The selection process and surgical procedures employed have, to date, not been standardized.

**Methods:**

A comprehensive 58-question web-based survey was developed with a focus on DBS referral practices and peri-operative management. The survey was distributed to the Parkinson’s Foundation Centers of Excellence, members of the International Parkinson’s Disease and Movement Disorders Society, and the Parkinson Study Group (Functional Neurosurgery Working Group) between December 2015 and May 2016.

**Results:**

There were 207 individual respondents (20% response rate) drawn from 59 countries and 6 continents, of whom 64% received formal training in DBS. Thirteen percent of centers reported that DBS could proceed despite a confidence level of < 50% for PD diagnosis. A case-based approach to DBS candidacy was applied in 51.3% of centers without a cut-off for levodopa-responsiveness. Surprisingly, 33% of centers regularly used imaging for diagnostic confirmation of idiopathic PD. Thirty-one percent of centers reported that neuropsychological evaluation did not affect DBS target selection. Approximately half of the respondents reported determination of DBS candidacy based on a multidisciplinary committee evaluation and 1/3^*rd*^ reported that a committee was used for target selection. Eight percent of respondents felt that psychosocial factors should not impact DBS candidacy nor site selection. Involvement of allied health professionals in the preoperative process was sparse. There was high variability in preoperative education about DBS outcome expectations. Approximately half of the respondents did not utilize a “default brain target,” though STN was used more commonly than GPi. Specific DBS procedure techniques applied, as well as follow-up timelines, were highly variable.

**Conclusion:**

Results revealed high variability on the best approaches for DBS candidate selection, brain target selection, procedure type, and postoperative practices. Cognitive and mood assessments were underutilized. There was low reliance on multidisciplinary teams or psychosocial factors to impact the decision-making process. There were small but significant differences in practice across global regions, especially regarding multidisciplinary teams. The wide variability of responses across multiple facets of DBS care highlights the need for prospective studies to inform evidence-based guidelines.

## Introduction

Parkinson’s disease (PD) is a progressive neurodegenerative disorder with both motor and non-motor symptoms and medical and surgical treatment options (Hughes et al., 1992; Postuma et al., 2015). The incidence of PD in the United States doubled between 1997 and 2017 (Collaborators et al., 2020). It has been estimated that there will be 1.64 million cases by 2037 (Yang et al., 2020). Although there are many approved medications for PD symptoms, select patients may require deep brain stimulation (DBS) surgery (Lozano et al., 2019; Ramirez-Zamora et al., 2019). DBS has been recognized as a treatment of choice for specific symptoms (tremor, dyskinesia, on-off fluctuations, off time) by several national and international guideline committees and expert consensus. Accordingly, DBS has been included in several professional society best-practices recommendations (National Collaborating Centre for Chronic Conditions, 2006; Pahwa et al., 2006; Fox et al., 2011; Ferreira et al., 2013).

Deep brain stimulation evaluation practices have gradually evolved over the past three decades. The original practices were borrowed from the core evaluations formulated by consensus for CAPIT (Langston et al., 1992) and CAPSIT-PD, which were initially developed for PD tissue transplantations (Defer et al., 1999). Initially, published “surgical” referral criteria were quite stringent, including proposing preoperative hospitalization in some instances (Broggi et al., 2003). In contrast, modern practices are such that most preoperative evaluations are completed in the outpatient setting.

Multiple groups have reported on their expert approaches (Pinter et al., 1999; Lang and Widner, 2002; Abboud et al., 2014) and posited exclusion criteria for DBS (Lopiano et al., 2002). Practices have been reported to vary widely across DBS centers in the areas of preoperative evaluation, candidate selection, brain target selection, and procedural techniques. The variability in DBS practices has limited generalizability in the extrapolation of DBS outcomes.

The current study utilized a comprehensive survey-based approach in collaboration with the International Parkinson’s Disease and Movement Disorders Society (MDS), the Parkinson’s Foundation Centers of Excellence (PF COE), and the Parkinson Study Group (PSG) Functional Neurosurgery Working Group. The study was international and aimed to uncover the variations in global DBS practices to inform future prospective outcome-directed research on DBS practices.

## Methods

A 58-question web-based survey (Supplementary File 1) on global DBS practice(s) was constructed. The survey focused on various aspects of the DBS referral pathway, including: initial referral mechanism, indications for DBS, adequacy of medication trials, method(s) of neuropsychiatric and neuropsychological evaluation, use and members of a multidisciplinary screening committee, brain target site selection, intra-, and postoperative imaging as well as postoperative management.

Questions regarding DBS referral and peri-operative management were formulated by a consensus of six practicing DBS experts at three centers. Discrepancies were addressed by consensus discussion among survey authors. The survey was distributed between December 17, 2015, and May 28, 2016, to the PF COEs, the MDS Functional Neurosurgery Committee members, and PSG functional neurosurgery working group members. An online survey system was used, with only one response from each participating DBS center permitted. When more than one response from a center was received, the authors identified a single representative response, typically the response from the practitioner’s response with the most years of experience in DBS. Results were tabulated and presented as a choice probability of response (denominator as the total number of question respondents). The complete dataset is available upon request to the corresponding author. Ethical review and approval was not required for the study on human participants in accordance with the local legislation and institutional requirements. Written informed consent from the participants was not required to participate in this study in accordance with the national legislation and the institutional requirements.

## Results

### Respondent Demographic Information

**FIGURE 1:**
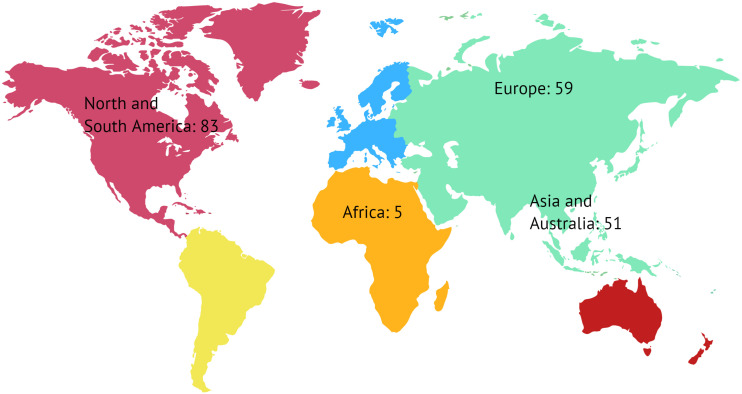
The survey had respondents from 4 regions representing 6 continents and 59 countries.

There were 207 individual respondents (20% of the sample) from 59 countries across six continents (Figure 1). Fifty-eight (58%) of respondents classified themselves as movement disorders neurologists (MDN) and 15% as neurosurgeons (Supplementary File 2). The average center experience for DBS surgery was 11.3 years (range: < 1 year to 32 years) and the average monthly number of surgeries was 3.3 (range: 0–15). Sixty-four percent of respondents received formal training in DBS (126/197), and 62% (78/126) reported a DBS manufacturer (i.e., industry) assisted in some role in their training (e.g., course).

### Referral Pathway

There were 91.5% (172/188) of respondents who responded that their center required an MDN evaluation before DBS surgery. Referrals directly to surgery could be made by general neurologists or outside MDNs in 11.2% (21/188) and 19.7% (37/188) of the sample, respectively, without evaluation by an internal neurologist or multidisciplinary committee. Sixty-seven percent (126/188) accepted self-referrals and 66.5% (125/188) accepted referrals from non-neurologists. About 50% (77/188) of respondents reported participating in direct-to-patient advertising for DBS surgery services.

### Pre-surgical Evaluation – Diagnosis

Responding centers reported 7.2 DBS referrals (range: < 1 to 42) and conducting an average 3.3 DBS procedures (range: < 1 to 15) a month. Besides PD, 83.5% (142/170), 79% (134/170), 70.6% (120/170) and 37.6% (64/170) of respondents reported performing DBS procedures for essential tremor, generalized dystonia, focal or segmental dystonia and Tourette’s syndrome/tics, respectively. Several other indications were also reported. Thirty-three percent (56/170) of respondents reported the use of functional imaging (including DaT SPECT imaging, PET, etc.) to confirm the diagnosis of idiopathic PD. Thirteen percent (22/170) of centers proceeded with DBS with a diagnostic confidence level of PD at ≤ 50%.

### Pre-surgical Evaluation – Medication Trials

**FIGURE 2:**
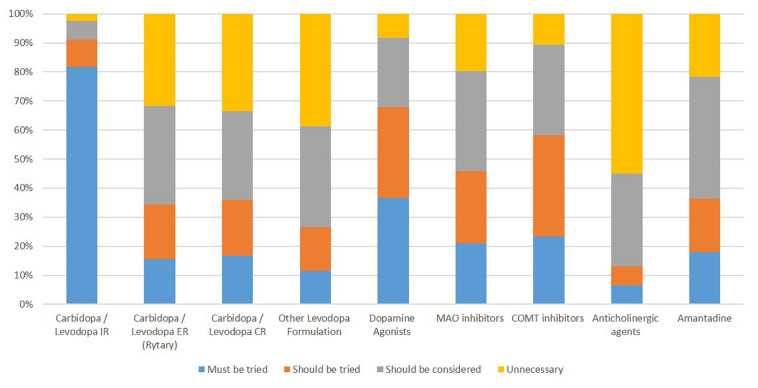
Percentage of respondents reporting pharmacotherapy use prior to Deep Brain Stimulation for Parkinson’s disease.

Approximately 93% (147/158) of respondents reported a process for determining the adequacy of pharmacotherapy before surgery. Almost half of the respondents (78/158) considered candidacy for intestinal gel-based levodopa (Duopa^TM^) simultaneously with DBS during the pre-surgical evaluation. 82% (129/158) felt immediate-release carbidopa/levodopa *must* be tried, while only 2.5% (4/158) felt it was unnecessary (see Figure 2 for details). The majority (86%) agreed that DBS should be considered if fluctuations were present despite dosing at least 5–6 times daily. While 18% (28/158) felt that there should be no minimum disease duration for consideration of DBS, 81% (128/158) felt it should be at least 3–4 years and 6.3% (10/158) felt that it should at least be seven years. Ninety percent (143/158) reported an OFF-ON Levodopa challenge as part of their DBS evaluation. A post-levodopa improvement on the Unified Parkinson Disease Rating Scale (UPDRS) or MDS-UPDRS of > 50% or > 33% were required in 15.2% (24/158) and 37.3% (59/158) of respondents, respectively. In contrast, 51.3% (83/158) of respondents reported a case-based approach without absolute cut-off of levodopa response.

### Pre-surgical Evaluation – Non-motor Features

Less than half the respondents (71/147) used specific, absolute cut-offs for a cognitive screen. Notably, 12.2% (18/147) respondents reported no formal neuropsychological evaluation required before DBS surgery. A formal neuropsychological evaluation was performed only if a cognitive screen suggested dysfunction at 68 (out of 147, 46.2%) centers. Suicidal ideation was not routinely assessed by 15.6% (23/147) of respondents.

There were questions to explore how the preoperative evaluation influenced decision-making regarding brain target or whether bilateral leads would be implanted simultaneously or staged. In 36% (53/147) of responses, mood evaluation never affected DBS target selection. In 30.6% (45/147) of responses, neurocognitive evaluation never affected DBS target selection (Supplementary File 2). Twenty-four percent (35/147) of respondents reported that procedures were never staged. Mood or neurocognitive evaluations would not have affected the decision to stage DBS in 49% (72/147) and 42.2% (62/147) of respondents, respectively (Supplementary File 2).

### Pre-surgical Evaluation – Rehabilitative and Psychosocial

Allied health professionals and rehabilitation staff were involved in the minority of preoperative evaluations: physical therapy (PT) 48%, occupational therapy (OT) 23.6%, speech therapy (SLP) 38.2%, social work 20.8%, case managers 13.2%, and registered nurses (34%, total number of respondents = 144). Eighty-five percent (124/144) of the responding centers’ evaluated psychosocial support and socioeconomic factors before DBS surgery, and only 7.6% (11/144) of respondents felt that these factors never affected DBS candidacy or site selection. On a question with multiple answers allowed (total number of respondents = 144), respondents reported that patients learned about DBS outcomes expectations from a variety of sources, including the referring neurologist/physician (40.3%), group seminar (27%), MDN (93%), a neurosurgeon (82%), psychiatrist (12.5%), neuropsychologist (28.5%) and registered nurse (32.6%).

### DBS Committee and Decision

**FIGURE 3:**
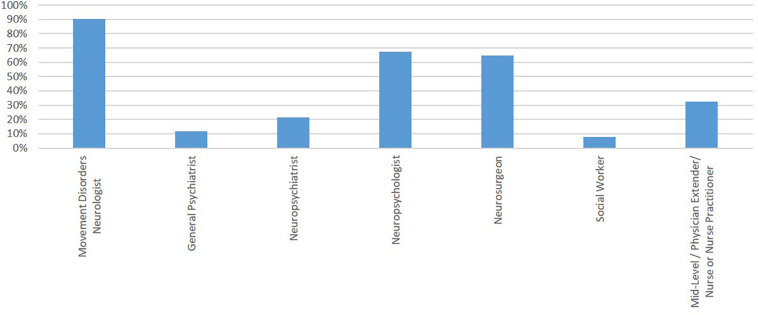
Team members reported as a part of the “required” preoperative evaluation. Y-axis represents the percentage of respondents reporting involvement of that team member.

Respondents considered various team members to be part of the “required” preoperative evaluation, though more and different specialists were variably available for evaluation (Figure 3). Ultimately, the candidacy for DBS for PD was determined by a DBS committee (46.5%; 67/144), MDN alone (18.7%; 27/144), MDN and neurosurgeon *without* a DBS committee (24.3%; 35/144), or by the neurosurgeon alone (10.4%; 15/144) across respondents. Likewise, the DBS target and procedure type was determined by a DBS committee (36.8%; 53/144), a MDN alone (13.2%; 19/144), an MDN and a neurosurgeon *without* a DBS committee (32%; 46/144), or a neurosurgeon alone (18%; 26/144). The final decision to proceed with DBS could be made via consensus-building (80%; 115/144), a veto by MDN (13.2%; 20/144), a veto by a neurosurgeon (16.6%; 24/144), a decision-making tool (1.4%; 2/144) or another modality (3.5%; 5/144).

### DBS Procedure

**FIGURE 4:**
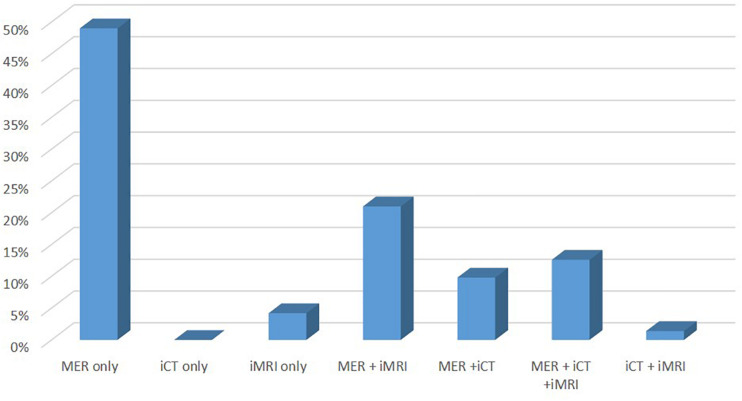
The intra-operative technique reported to be utilized to evaluate and/or to confirm micro-macro-electrode position. Y-axis represents percentage of respondents reporting use of that technique. More than one technique may be used in a given institution. Abbreviations: MER, microelectrode recording; iCT, intraoperative computed tomography; iMRI, intraoperative magnetic resonance imaging.

The following intra-operative technique(s) were reported to be utilized to evaluate or to confirm micro- or macro-electrode position (total number of respondents = 143): Microelectrode recording or MER (91.6%), Image guidance-CT (25.2%), and image guidance-MRI (40.5%) (Figure 4). Several centers performed more than one type of lead localizing procedure. 49% of the respondents reported using MER only. 9.8% used MER with iCT, 21% used MER with iMRI, and 12.6% used all three modalities (MER, iCT, and iMRI). No respondent reported using iCT alone, whereas 4.2% of respondents reported using iMRI alone. 1.4% reported using iCT and iMRI without MER use.

Via multiple response questions with more than one response allowed, MER recording and analysis were performed by a neurologist (62.9%), a neurosurgeon (37%), a physiologist (30.8%), or others (7%). Relatedly, the preoperative and peri-operative stereotactic planning for the DBS target was reported as performed by a neurologist (26.6%), a neurosurgeon (92.3%); a physiologist (7%), a radiologist (4.9%), or by a representative from a medical device company (9%).

**FIGURE 5:**
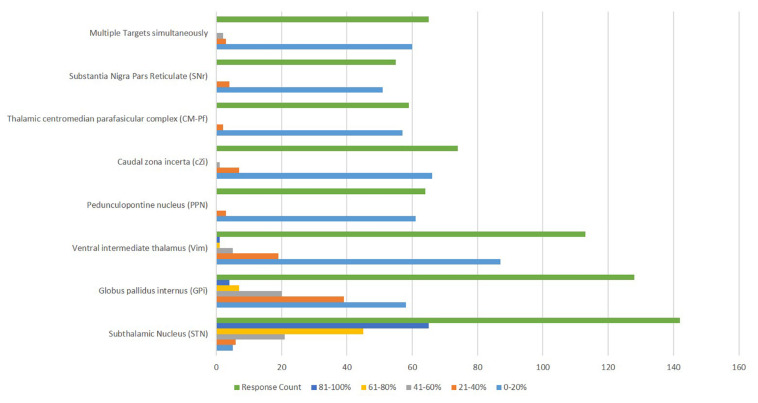
Deep Brain Stimulation targets reported to be utilized for the management of Parkinson’s disease.

Approximately half of the respondents (51%) did not utilize a “default target.” STN was used more commonly than GPi as a target. Few centers used alternative targets (Figure 5). Many centers (45.4%) targeted STN for 81-100% of PD cases, while only 2.8% (65/143) targeted GPi and 0.7% (1/143) targeted Vim with that frequency. PPN was reported to be used 21-40% of the time by three centers and cZI by seven centers at that proportion of cases. Other targets were also pursued in some participating centers.

### Post-implantation and Follow-Up Care

Postoperative imaging was obtained within 24 h by 66.4% (95/143) of centers, while 7.7% (11/143) of centers did not routinely obtain postoperative imaging. Overall, CT was used by 73.4% (105/143) and MRI by 36.4% (52/143) of respondents. No respondent reported using ventriculography. Feedback to the referring physician about clinical efficacy was provided by the MDN (78.3%; 112/143), a neurosurgeon (14.7%; 21/143), or was not provided (7%; 10/143). Seventy-three percent (105/143) of respondents routinely evaluated mood or cognitive disability/sequelae postoperatively. While a pre-specified schedule for follow up was reported by 43.3% (62/143) of respondents, 12% (17/143) reported no specific routine follow-up. The following services did not participate in the routine postoperative evaluations in nearly 40% (57/143) of centers: PT, OT or SLP, social workers, case managers, psychiatrists/neuropsychiatrists neuropsychologists. Only 31% (44/143) of centers had a formal DBS specific mortality-morbidity conference.

### Regional Variability

The regional variability of key DBS practices are as follows. The African region only had five responses limiting further data exploration (Table 1).

**TABLE 1:** Regional variability in Deep Brain Stimulation practices in Parkinson’s disease.

	North and South America	Asia and Australia	Europe	Africa
Number of respondents	83	51	59	5
Movement disorders neurologists respondents	57%	53%	65.5%	20%
Center experience (years)	11.7	9.4	12.4	2
Formal DBS training	67%	63%	67%	20%
Number of DBS referrals per month for PD	9	5	6.8	1.2
Number of DBS procedures per month for PD	4.3	2.2	2.7	0.2
Assessment of pharmacotherapy adequacy prior to DBS	97%	97%	91%	50%
No cut-off for disease duration prior to DBS	22%	16%	9%	0%
No absolute cut-off for motor improvement for DBS consideration	54%	57%	41%	0%
No formal neuropsychological testing prior to DBS	7.5%	5%	5%	50%
Default target for DBS in PD	43%	56%	50%	50%
DBS candidacy decided based on a multidisciplinary committee	40%	69%	69%	50%
Use of intra-operative MER in the institution	89%	100%	90%	100%
Pre-specified schedule clinic follow up post DBS	42.4%	31.2%	56.4%	100%

*Abbreviations: DBS, Deep Brain Stimulation; PD, Parkinson’s disease; MER, Microelectrode recording.*

#### Respondent Demographic Information

##### North and South America

The average center experience of DBS surgery was 11.7 years. Sixty-seven percent (51/76) of respondents received formal training in DBS.

##### Asia and Australia

The average center experience of DBS surgery was 9.4 years. Sixty-three percent (31/49) of respondents received formal training in DBS.

##### Europe

The average center experience of DBS surgery was 12.4 years. Seventy-one percent (41/58) of respondents received formal training in DBS.

#### Referral Pathway and Pre-surgical Evaluation – Diagnosis

##### North and South America

For 92% (67/73) of respondents, an MDN evaluation was necessary before DBS surgery.

Responding centers reported receiving an average number of 9 DBS referrals and conducting an average of 4.3 DBS procedures a month.

##### Asia and Australia

For 91.5% (43/47) of respondents, an MDN evaluation was necessary before DBS surgery.

Responding centers reported receiving an average number of 5 DBS referrals and conducting on an average 2.2 DBS procedures a month.

##### Europe

For 98% (53/54) of respondents, an MDN evaluation was necessary before DBS surgery.

Responding centers reported receiving an average number of 6.8 DBS referrals and conducting on an average 2.7 DBS procedures a month.

#### Pre-surgical Evaluation - Medication Trials

##### North and South America

Whether a trial of levodopa/carbidopa immediate must be tried before DBS for PD was endorsed by 87% (*n* = 60), whereas none felt it was unnecessary. While 22% (15/69) felt that there should be no minimum disease duration for DBS consideration for PD, 6% (4/69) felt that it should be more than seven years. While 7% (5/69) required at least 50% improvement on UPDRS or MDS-UPDRS before proceeding with DBS, 54% (37/69) reported a case-based approach with no absolute cut-off.

##### Asia and Australia

Whether a trial of levodopa/carbidopa immediate must be tried before DBS for PD was endorsed by 81% (30/37), whereas none felt it was unnecessary. While 16% (6/37) felt that there should be no minimum disease duration for DBS consideration for PD, 8% (3/37) felt that it should be more than seven years. While 11% (4/37) required at least 50% improvement on UPDRS or MDS-UPDRS before proceeding with DBS, 57% (21/37) reported a case-based approach with no absolute cut-off.

##### Europe

Whether a trial of levodopa/carbidopa immediate must be tried before DBS for PD was endorsed by 75% (33/44), whereas 4.5% (2/44) felt it was unnecessary. While 9% (4/44) felt that there should be no minimum disease duration for DBS consideration for PD, 7% (3/44) felt that it should be more than seven years. While 29.5% (13/44) required at least 50% improvement on UPDRS or MDS-UPDRS before proceeding with DBS, 41% (18/44) reported a case-based approach with no absolute cut-off.

#### Pre-surgical Evaluation – Non-motor Features

##### North and South America

98.5% (66/67) of respondents reported cognitive symptoms routinely screened pre-DBS. Only 7.5% (5/67) of respondents reported no requirement of formal neuropsychological evaluation before DBS surgery. 43% (29/67) of respondents reported using a default brain target for DBS for PD.

##### Asia and Australia

Ninety five percentage (33/34) of respondents reported cognitive symptoms routinely screened pre-DBS. Only 5% (10/34) of respondents reported no requirement of formal neuropsychological evaluation before DBS surgery. 56% (19/34) of respondents reported using a default brain target for DBS for PD.

##### Europe

Ninety five percentage (38/40) of respondents reported cognitive symptoms routinely screened pre-DBS. Only 5% (2/40) of respondents reported no requirement of formal neuropsychological evaluation before DBS surgery. 50% (20/40) of respondents reported using a default brain target for DBS for PD.

#### DBS Committee and Decision

##### North and South America

A committee determined DBS candidacy for PD in 40%, brain target, and procedure type in 30% of centers. The final decision to proceed with DBS was established by consensus building in 82% (55/67).

##### Asia and Australia

A committee determines DBS candidacy for PD in 69%, brain target, and procedure type in 22% of respondents. The final decision to proceed with DBS was established by consensus building in 75% (24/32).

##### Europe

A committee determines DBS candidacy for PD in 69%%, brain target, and procedure type in 56.4% of respondents. The final decision to proceed with DBS was established by consensus building in 87% (34/39).

#### DBS Procedure

##### North and South America

The following intra-operative technique(s) were reported to be utilized to evaluate and confirm micro-macro-electrode position (total number of respondents = 67): Microelectrode recording or MER (89%; 60/67), Image guidance-CT (28.3%; 19/67), and image guidance-MRI (42%; 28/67).

In a question with multiple options allowed (total number of respondents = 67), the recording and analysis of MER was reported to be performed by the neurologist (54%; 36/67), neurosurgeon (40.3%; 27/67), physiologist (33%; 22/67) and others (6%; 4/67).

In a question with multiple options allowed (total number of respondents = 67), the preoperative and peri-operative stereotactic planning for the selected DBS target was reported to be performed by the neurologist (21%; 14/67), neurosurgeon (91%; 61/67); physiologist (7.5%; 5/67) and radiologist (3%; 2/67).

##### Asia and Australia

The following intra-operative technique(s) were reported to be utilized to evaluate and confirm micro-macro-electrode position (total number of respondents = 32): Microelectrode recording or MER (100%; 32/32), Image guidance-CT (16%; 5/32), and image guidance-MRI (34.4%; 11/32).

In a question with multiple options allowed (total number of respondents = 32), the recording and analysis of MER were reported to be performed by the neurologist (78%; 25/32), neurosurgeon (44%; 14/32), and physiologist (28%; 9/32).

In a question with multiple options allowed (total number of respondents = 32), the preoperative and peri-operative stereotactic planning for the selected DBS target was reported to be performed by the neurologist (44%; 14/32), a neurosurgeon (84%; 27/32); physiologist (9.4%; 3/32) and radiologist (6.2%; 2/32).

##### Europe

The following intra-operative technique(s) were reported to be utilized to evaluate and confirm micro-macro-electrode position (total number of respondents = 39): Microelectrode recording or MER (90%; 35/39), Image guidance-CT (26%; 10/39), and image guidance-MRI (41%; 16/39).

In a question with multiple options allowed (total number of respondents = 39), the recording and analysis of MER were reported to be performed by the neurologist (69%; 27/39), neurosurgeon (26%; 10/39), physiologist (31%; 12/39) and others (10%; 4/39).

In a question with multiple options allowed (total number of respondents = 39), the pre-operative and peri-operative stereotactic planning for the selected DBS target was reported to be performed by the neurologist (20.5%; 8/39), neurosurgeon (100%; 39/39); physiologist (2.6%; 1/39) and radiologist (7.7%; 3/39).

#### Post-implantation and Follow-Up Care

##### North and South America

Postoperative imaging was not obtained by 7.6% of centers routinely (unless there were unexpected signs or symptoms), and 9% reported no specific routine to follow-up with DBS check whenever needed or during PD follow-up visit.

##### Asia and Australia

Postoperative imaging was not obtained by 12.5% of centers routinely (unless there were unexpected signs or symptoms), and 22% reported no specific routine follow-up with DBS check whenever needed or during PD follow-up visit.

##### Europe

Postoperative imaging was not obtained by 2.6% of centers routinely (unless there were unexpected signs or symptoms), and 5% reported no specific routine follow-up with DBS check whenever needed or during PD follow-up visit.

## Discussion

The data from this global survey revealed variability in international DBS practice, including preoperative motor evaluation, preoperative non-motor evaluation, DBS decision-making, procedure type, and postoperative assessment of outcomes. The involvement of respondents from 59 countries, spread across six continents and the four regional sections (acknowledged by the International Parkinson’s disease and Movement Disorder Society) strongly supported the survey’s global intentions. While a survey-based methodology could be susceptible to several sources of bias, there were clear and expected areas of variability that warrant further inquiry.

One potential source of variability in DBS practice(s) is the wide variety of pathways through which providers receive training in the management of DBS patients. It was somewhat concerning that 36% of respondents reported *no* formal training in DBS during post-graduate, subspecialty training, or fellowship experience. DBS was first approved by the United States Food and Drug Administration in 1997 and even earlier in Europe. As the average duration of practice among respondents was 11.3 years (range < 1 to 32y), many respondents likely began managing DBS patients before widespread clinical use and training (movement disorders neurology or functional neurosurgery fellowships). However, these data did suggest that most respondents may have finished training within the last two decades. This would correspond to when DBS education should have likely been integrated into post-residency programs. Interestingly, a majority of respondents (62%) reported training by industry. Though device manufacturer-sponsored courses are valuable, most experts would agree that they should not be the main drivers of education in the field. There are three FDA- and CE-approved DBS manufacturers with 20 + companies in the DBS development pipeline internationally (DelveInsight’s, 2020). The involvement of industry as a primary source of DBS education will introduce a major source of variability in DBS practice given that each device manufacturer may emphasize different management principles (imaging-based, neurophysiology-based, segmented leads, etc.). Some of the heterogeneity in training may be related to restricted access to movement disorders training programs, though we did not explore this issue within our dataset. There is wide variability in the availability of training for DBS; for instance, the world’s second most populous country, India, has only 8-10 movement disorders fellowships and one functional neurosurgery training program, which is far less than what is needed (Zhang et al., 2020). Collectively, the data suggest that there may be space for improvement in the standardization of essential educational elements expected in DBS training. We also would advocate that the influence of industry education on trainees’ education should be more closely monitored.

The variability in diagnostic confirmation techniques for PD was particularly notable. The most recent clinical diagnostic criteria for PD supports a diagnosis of probable or clinically established PD, based mainly on history and physical examination (Postuma et al., 2015), with ancillary testing only necessary when there is an accompanying suspicion of a secondary cause of parkinsonism (Berg and Adler, 2018). Therefore, the use of functional imaging to confirm PD diagnosis by 33% of respondents was surprising and may be driven by the recent shift toward earlier DBS (Schuepbach et al., 2013; Hacker et al., 2020). On the other hand, 13 centers reporting proceeding with DBS for PD when diagnostic certainty was < 50% demonstrates that there is still high variability across centers regarding their degree of concern over diagnostic certainty. Lack of standardization to guide pre-DBS candidacy determination may lead to adverse outcomes (Mari, 2020).

The survey results offer insight into the decision-making processes employed at many DBS centers. A slight majority (51.3%) of centers used a patient-centered approach to candidacy and did not employ cut-offs for the degree of levodopa response (Kleiner-Fisman et al., 2006). The data supports a growing acknowledgment that using a 33% improvement in UPDRS or MDS-UPDRS Part III as a cut-off will limit DBS access, especially in patients with medication-refractory tremor (or other unique symptom profiles). We posit that the use of hard cut-offs on UPDRS scales may inadvertently exclude specific patients who may benefit from DBS, and systems of care should investigate the extent to which these criteria may inadvertently introduce an undue burden on clinicians and patients. Individual patient symptoms and expectations must be taken into consideration before making decisions pertaining to DBS surgery and assessing outcomes.

Rather than using strict cut-offs, consensus recommendations from DBS experts promote the use of a pre-DBS multidisciplinary team to review the motor, cognitive, psychiatric, and psychosocial status in the development of a risk-benefit estimate (Abboud et al., 2014; Higuchi et al., 2016; Akbar and Asaad, 2017). However, only half of the respondents reported using a multidisciplinary committee to determine DBS candidacy. Furthermore, 12.2% of respondents did not require neuropsychological evaluation, with 46.2% if deficits were uncovered mandating a conditional neuropsychological evaluation after a cognitive screening examination (Rothlind et al., 2015; Cernera et al., 2019; Kenney et al., 2020). The literature is evolving but supportive of the notion that brain target selection can impact cognitive or and mood outcomes following DBS (Okun et al., 2009; Rothlind et al., 2015; Kenney et al., 2020) and that baseline cognitive performance predicts post-DBS cognitive decline and quality of life (Odekerken et al., 2015; Kenney et al., 2020). The survey revealed room for potential improvement in utilizing multidisciplinary teams with patient-centered assessments, including neuropsychological and psychosocial function, rather than relying on strict rating scale cut-offs, permitting more inclusiveness for patients who may benefit from DBS.

The involvement of allied health professionals varied considerably across centers. More than half of respondents reported that routine physical therapy assessments were not utilized. The utility of preoperative PT assessments will warrant further study given that the types and severity of baseline gait and postural abnormalities could potentially inform the DBS team (and patient), particularly in postoperative gait and balance expectations (Nantel et al., 2012). Only 20.8% and 13% of respondents reported social workers or case managers’ were involved in the DBS preoperative process, though our survey did not quantitate why these professionals were not utilized. With training in assessment of care partner burden and psychosocial challenges, social workers or case managers might better prepare the DBS team’s expectations for having a patient and care partner who facilitate successful DBS therapy, or they might help to identify and mitigate social determinants of health in order to optimize outcomes. Understanding the barriers or reluctance to use these allied health professionals in perioperative DBS management will be a potential area for future study.

The preoperative education on outcome expectations was highly variable among our respondents, with only about 1/4^*th*^ (27%) of centers using a formalized educational format such as a seminar or lecture to supplement education from neurologists (40.3%) and neurosurgeons (82%). Educational programs such as ParkEduStim might help to align patient expectations with potential results from surgery (Valérie Fraix and Schmitt, 2021). Patient and care partner expectation management will be integral to achieving patient satisfaction with DBS and other surgical procedures (Maier et al., 2013; Knoop et al., 2017). Whether the presence or absence of structured DBS educational programs in the preoperative evaluation changes decision-making at the patient or provider level is unclear, but current evidence suggests that it increases patient satisfaction. In a recent retrospective analysis of DBS cases referred for second opinions, nearly half of the “unsatisfied patients” complained of symptoms that DBS could not address, including cognitive impairment, imbalance, dysarthria, and dysphagia (Kluger et al., 2011). Use of a formal education seminar, internally or directed to reliable external sources (Parkinsons Foundation, 2020) may lead to more concurrence between patient and provider expectations. Whether cultural issues drove the gap in education and management of preoperative expectations, availability of services or other factors was unclear.

The brain target variability matches the literature suggesting STN or GPi targets can be used for PD (Deuschl et al., 2019). However, some respondents predominantly used a specific brain target (45.4% of centers used STN in 81–100% of cases). Approximately half of the respondents (51%) did not utilize a “default target,” though STN was used more commonly than GPi. We suspect technical considerations such as familiarity with MER, access to intraoperative imaging, surgical experience, or center-specific outcome trends can potentially influence target choice. Interestingly, some centers reported a high frequency of implanting alternative targets such PPN (21–40% of cases at three centers) or cZI (21-40% at seven centers). Since there are many factors in choosing a DBS target, our results were unsurprising.

We stratified responses to the survey by region (excluding Africa, which only provided five responses), and by analyzing the data in this fashion, we observed only small inter-region variability across most questions. Centers located in the Americas tended to be less likely to use a specific cut-off for disease duration for DBS candidacy (22% of centers in the Americas, 16% in Asia/Australia, versus 9% in Europe). American region centers were also less likely to decide on DBS candidacy based on a multidisciplinary committee (40% of centers in the Americas, 69% in Asia/Australia, versus 69% in Europe. We speculate that payor systems or cultural norms may have driven these differences; however, we could not uncover the rationale from the dataset.

Centers in the Americas had a lower rate (43%) of a “default target” as compared to Europe (50%), Asia (55%), and Australia (67%). We speculate that this could be sequelae of differences in outcomes between the two largest trials comparing brain targets. The North American trial (Follett et al., 2010) showed equipoise regarding motor symptom outcomes when comparing STN and GPi DBS, while the Dutch/European trial favored STN for the secondary outcome of motoric benefit (Odekerken et al., 2013). Thus, using a default target might seem more appropriate if greater weight is given to the latter trial. Only 40% of the Americas’ centers reported using a multidisciplinary committee for decision-making, while 69% of centers in both Asia/Australia and Europe used a committee. We do not know how many solo or small group DBS practices exist in the Americas, especially North America when compared to other countries. We suspect healthcare systems outside of the Americas’ to more commonly use centralized hubs of healthcare (Ridic et al., 2012), potentially providing more consistent access to a multidisciplinary team.

Our study was not without limitations, the foremost of which is that surveys are usually susceptible to selection bias. To counteract this issue, we attempted to reach as many providers as possible by dissemination through the International Parkinson’s disease and Movement Disorders society and other major organizations. While our survey probably over-represents larger or academic DBS centers, there were many respondents with low volumes of only 1-2 surgeries per month, suggesting we also captured small and mid-size programs. Additionally, surveys can also be susceptible to information bias based on the question’s wording. We developed the survey with input from six experienced providers, including representatives from psychiatry, neurology, and neurosurgery, to address this issue. Another issue was duplicate responses from the same surgical center. We addressed this issue by only considering a single response per center, and we prioritized based on the respondent’s experience. Our survey focused on DBS practices and did not inquire about the availability, expertise or utilization of stereotactic lesioning because (a) we wanted to minimize attrition by keeping the survey as short as possible, (b) lesioning is widely used but perhaps not completely overlapping with DBS centers so a parallel question set would have been required, and (c) the risk assessment performed for DBS is potentially different than invasive or non-invasive lesioning procedures, and thus would have required separate responses.

In summary, the survey results reflect wide variability and a lack of consensus in many critical areas of PD DBS practice. Though variability can be important to improve surgical procedures, we would argue that the presentation of this and other future datasets may be useful in guiding the field toward better outcomes. The dialog should include discussing issues where a more homogenous approach across centers may improve overall outcome(s). Finally, we propose that similar surveys, perhaps coupled with outcome registries, be circulated periodically as a monitoring tool for the DBS field.

## Data Availability Statement

The raw data supporting the conclusions of this article will be made available by the authors, without undue reservation.

## Ethics Statement

Ethical review and approval was not required for the study on human participants in accordance with the local legislation and institutional requirements. Written informed consent for participation was not required for this study in accordance with the national legislation and the institutional requirements.

## Author Contributions

AM did the analysis or interpretation of data, drafting of the manuscript, and critical revision of the manuscript for important intellectual content. AB did the acquisition, analysis or interpretation of data, and critical revision of the manuscript for important intellectual content. MO did the analysis or interpretation of data, and critical revision of the manuscript for important intellectual content. ZM did the concept, acquisition, analysis or interpretation of data, critical revision of the manuscript for important intellectual content, and supervision. KM did the acquisition, analysis or interpretation of data, critical revision of the manuscript for important intellectual content, and supervision. All authors contributed to the article and approved the submitted version.

## Conflict of Interest

The authors declare that the research was conducted in the absence of any commercial or financial relationships that could be construed as a potential conflict of interest.
