# Supplementary material for: Global Variability in Deep Brain Stimulation Practices for Parkinson’s Disease
Source: Front Hum Neurosci. 2021 Mar 31;15:667035. doi: 10.3389/fnhum.2021.667035 (PMC8044366; doi:10.3389/fnhum.2021.667035)
Supplement: Supplementary file 1 [file Data_Sheet_1.PDF]

Deep Brain Stimulation Center Survey

Welcome to My Survey

Thank you for participating in our survey. This survey is in collaboration with the National Parkinson Foundation, Parkinson Disease Study Group and the Movement Disorders Society.

This survey aims to better understand how various Physicians and Deep Brain Stimulation Centers handle the referral, pre-operative, peri-operative and post-operative care of Parkinson Disease patients undergoing evaluation for Deep Brain Stimulation. The questions are largely structured as multiple choice or YES-NO. For questions with instructions to "select all that apply", you do not need to select an answer if none apply.

Academic Centers - As we are conducting this Survey as a*per center* evaluation, please pick the choice which is most representative of your group.

PARKINSON'S FOUNDATION

Deep Brain Stimulation Center Survey

Respondent Contact Information

Please enter your contact information below. This information will only be used if we wish to follow-up with you to clarify your response.

1. Address

Name

State/Province

ZIP/Postal Code

Country

Email Address

Respondent Demographic Information

2. We will ask specific, sometimes rather technical, questions pertaining to DBS practices. We would like to know if the responses reflect your individual choices or those of your center. You can make your selection now but may decide to change as you go through these questions. You will have an opportunity to confirm your choice at the end. Please select one of the following:

- ◯ I am responding on behalf of our center / team / group practice and these choices reflect consensus among our team.
- ◯ I am responding as an individual, it is possible that colleagues in my group / team / center would respond differently.

3. Please indicate your specialty / training

- ◯ Neurologist (Non-Movement Disorders Trained)
- ◯ Movement Disorders Neurologist (MDN)
- ◯ Neurosurgeon
- ◯ Psychiatrist
- ◯ Neuropsychologist
- ◯ Resident / Fellow
- ◯ Nurse Practitioner (NP or ARNP)
- ◯ Physician Assistant (PA-C)

4. How many years has DBS been performed at your center?

[ ]

5. Did you receive specific training for DBS during your fellowship?

- ◯ Yes
- ◯ No

6. Did you receive training by a DBS Manufacturer (Medtronic, St. Jude etc.)?

- ◯ Yes
- ◯ No

Referral Pathway

Please provide details on your center's DBS Referral Pathway

7. Is an evaluation by a Movement Disorders Neurologist (MDN) *necessary* prior to having DBS Surgery?

◯ Yes

◯ No

8. Can patients referred by an outside General Neurologist go *directly* to surgery without evaluation by a Movement Disorder Neurologist and/or your Center's multidisciplinary DBS Committee review?

◯ Yes

◯ No

9. Can patients referred by an outside Movement Disorders Neurologist go *directly* to surgery without evaluation by an internal MDN and/or your Center's multidisciplinary review?

◯ Yes

◯ No

10. Does your DBS Center accept self-referrals for DBS evaluation?

◯ Yes

◯ No

11. Does your DBS Center accept referrals from non-Neurologists for DBS Evaluation?

◯ Yes

◯ No

12. Does your Center participate in direct-to-patient advertising for Deep Brain Stimulation?

◯ Yes

◯ No

Pre-surgical Evaluation - Diagnosis

Please elaborate on how your Center evaluates patients pre-surgically

13. Does your Center utilize any structured pre-surgical, decision-making tools to determine candidacy for DBS? Select all that apply. (You may skip this question if you do not use a decision-making tool)

- [ ] FLASQ-PD
- [ ] STIMULUS
- [ ] Other

14. What is your center's *average* number of referrals for DBS evaluation for Parkinson Disease per month?

____

15. What is your center's *average* number of DBS surgeries for Parkinson Disease per month?

____

16. Besides Parkinson Disease, for what other diagnoses / conditions does your center use DBS (including clinical and research indications)?

- [ ] Essential Tremor
- [ ] Generalized Dystonia
- [ ] Focal or Segmental Dystonia
- [ ] Tic Disorder / Tourettes

Other or Investigatory Indications (please list)

____

17. **Which evaluations does your center routinely use to confirm the diagnosis of idiopathic Parkinson's disease? Please select all that apply.**

- [ ] Routine History & Physical
- [ ] Structured History & Physical (UK Brain Bank Criteria)
- [ ] OFF-ON Testing
- [ ] CT
- [ ] MRI
- [ ] Functional Imaging (DaTSCAN, PET, fMRI etc.)

18. In the opinion of your DBS Committee, what is the *lowest level of confidence* on diagnosis of Idiopathic Parkinson Disease permissible to proceed with a DBS evaluation?

<10%	11-20%	21-30%	31-40%	41-50%	51-60%	61-70%	71-80%	81-90%	>90%
○	○	○	○	○	○	○	○	○	○

Pre-surgical Evaluation - Medication Trials

Please clarify how your Center determines whether a potential DBS candidate has had an adequate medication trial

19. Does your clinical pathway / multidisciplinary review include a determination about pharmacotherapy adequacy and/or provide recommendations for further medication trials prior to issuing a verdict about DBS candidacy?

- ○ Yes
- ○ No

20. Does your center consider candidacy for Intestinal Levodopa (Duopa) simultaneously with DBS during pre-surgical evaluation?

- ○ Yes
- ○ No

21. Prior to proceeding with DBS for Parkinson Disease, which of the following agents must be tried, should be tried or at least considered with medical justification for avoiding a trial

	Must be tried	Should be tried	Should be considered	Unnecessary
Carbidopa / Levodopa Immediate Release	◯	◯	◯	◯
Carbidopa / Levodopa Extended Release (Rytary)	◯	◯	◯	◯
Carbidopa / Levodopa Controlled Release (CR)	◯	◯	◯	◯
Other Levodopa Formulation (orally dissolving, intestinal etc.)	◯	◯	◯	◯
Dopamine Agonists (Pramipexole, Ropinirole etc)	◯	◯	◯	◯
MAO inhibitors (eg Rasagaline etc)	◯	◯	◯	◯
COMT inhibitors (eg Entacapone etc)	◯	◯	◯	◯
Anticholinergic agents (eg Trihexyphenidyl)	◯	◯	◯	◯
Amantadine	◯	◯	◯	◯

22. What frequency of dopaminergic medication usage, in doses per day, typically warrants DBS if fluctuations are still present (including overnight doses)?

<3	3-4	5-6	7-8	>9
◯	◯	◯	◯	◯

23. What is the minimum duration of Parkinson Disease before DBS should be considered?

◯ There is no minimum duration of disease

◯ <2 years

◯ 3-4 years

◯ 5-6 years

◯ 7-10 years

◯ >10 years

24. How determined is the average patient to obtain / proceed with DBS?

Not motivated	Somewhat motivated	Very motivated	Determined	Very determined
○	○	○	○	○

25. Is an in-house (at your DBS Center) OFF-ON Levodopa challenge required prior to DBS consideration?

○ Yes

○ No

26. Are your center's OFF-ON Levodopa challenges video recorded?

○ Yes

○ No

27. Do you have a *strict cut-off(s)* of improvement before proceeding with DBS? Please select all that apply.

☐ Case-Based

☐ 30 point improvement on UPDRS or MDS-UPDRS

☐ 33% improvement on UPDRS or MDS-UPDRS

☐ 50% improvement on UPDRS or MDS-UPDRS

☐ Other

Pre-Surgical Evaluation - Non-Motor Features

Please clarify how your center handles the evaluation of non-motor and neuropsychiatric aspects of the DBS evaluation

28. How do you screen mood symptoms during initial DBS evaluation? Please select all that apply.

- ☐ Mood symptoms are not routinely screened
- ☐ Clinical Interview
- ☐ Neuropsychiatric Inventory (NPI)
- ☐ Parkinson Disease Questionnaire (PDQ-39)
- ☐ Subjective (Patient-Rated) Mood Questionnaire (QIDS, Beck, Zung etc)
- ☐ Objective (Clinician-Rated) Mood mood questionnaire (MADRS / HDRS etc)

29. Is suicidal ideation, specifically, assessed before and after DBS evaluation?

- ○ Yes
- ○ No

30. Who evaluates all patients undergoing DBS evaluation, regardless of prior psychiatric history or symptoms?

- ☐ Movement Disorders Neurologist
- ☐ General Psychiatrist
- ☐ Neuropsychiatrist
- ☐ Neuropsychologist
- ☐ Neurosurgeon
- ☐ Social Worker
- ☐ Mid-Level / Physician Extender
- ☐ Nurse -- RN or NP

31. Evaluation by what other specialists is available, if needed, for neuropsychiatric and cognitive symptoms at your DBS Center?

- [ ] General Psychiatrist
- [ ] Neuropsychiatrist
- [ ] Neuropsychologist
- [ ] Social Worker
- [ ] Psychotherapist or Mental Health Counselor
- [ ] Mid-Level / Physician Extender

32. How do you screen cognitive symptoms during initial DBS evaluation?

- [ ] Cognitive symptoms are not routinely screened *[If selecting, please do not choose any other option below]*
- [ ] Mini-Mental Status Examination (MMSE)
- [ ] Montreal Cognitive Assessment (MoCA)
- [ ] Dementia Rating Scale (DRS)
- [ ] Other or unlisted test
- [ ] Only evaluated during neuropsychological testing

33. Are specific, absolute, cut-offs utilized during cognitive screen?

- ○ Yes
- ○ No

34. Is a formal <u>Neuropsychological Evaluation</u> *required* before DBS Surgery?

- ○ Yes
- ○ No

35. Is a formal <u>Neuropsychological Evaluation</u> *conditional* based on cognitive screening?

- ○ Yes
- ○ No

36. Does your center generally use a "default" brain target for DBS?

- ○ Yes
- ○ No

37. If your center generally uses a "default" brain target for DBS, how often is DBS target selection affected by results of

	Never	<10%	11-25%	25-50%	51-75%	76-100%
Mood Evaluation	○	○	○	○	○	○
Neurocognitive Evaluation	○	○	○	○	○	○

38. How often are DBS procedures staged?

Never	<10%	11-25%	26-50%	51-75%	76-100%
○	○	○	○	○	○

39. How often are bilateral DBS procedures staged based on results of:

	Never	<10%	11-25%	26-50%	51-75%	76-100%
Mood Evaluation	○	○	○	○	○	○
Neurocognitive Evaluation	○	○	○	○	○	○

Pre-Surgical Evaluation - Rehabilitative & Psychosocial

Please clarify how your center works with Allied Professionals and assesses psychosocial aspects of DBS candidacy

40. Which (if any) Allied Professionals and Rehabilitation staff are routinely involved in all pre-operative evaluations of DBS candidacy? Please select all that apply. If you utilize none of the choices, you may skip this question.

- ☐ Physical Therapy
- ☐ Occupational Therapy
- ☐ Speech, Swallowing and Language Pathology
- ☐ Social Workers
- ☐ Case Managers
- ☐ Registered Nurse

41. Are psychosocial supports and socioeconomic factors routinely evaluated before DBS surgery?

- ◯ Yes
- ◯ No

42. How often do psychosocial and socioeconomic factors affect DBS candidacy or site selection?

Never	<10%	11-25%	26-50%	51-75%	76-100%
◯	◯	◯	◯	◯	◯

43. How does the patient learn about expectations for DBS surgical outcomes? Select all that apply

- ☐ Referring Neurologist / Physician
- ☐ Group Seminar / Lecture
- ☐ Movement Disorders Neurologist
- ☐ Neurosurgeon
- ☐ Psychiatrist
- ☐ Neuropsychologist
- ☐ Registered Nurse

DBS Committee and Decision

Please describe how your Center handles the final decision to proceed with surgery

44. Who, ultimately, determines the candidacy for DBS for Parkinson Disease at your center?

- ◯ Referring Neurologist / Physician
- ◯ Movement Disorder Neurologist
- ◯ Neurosurgeon
- ◯ Movement Disorder Neurologist & Neurosurgeon *only* (without DBS Committee)
- ◯ DBS Committee

45. Who, ultimately, determines the brain target and procedure type (if options exist)?

- ◯ Referring Neurologist / Physician
- ◯ Movement Disorder Neurologist
- ◯ Neurosurgeon
- ◯ Movement Disorder Neurologist & Neurosurgeon *only* (without DBS Committee)
- ◯ DBS Committee

46. How is the decision to proceed with DBS established?

- ☐ Consensus Building
- ☐ Veto by Movement Disorders Neurologist
- ☐ Veto by Neurosurgeon
- ☐ Decision Making Tool (FLASQ-PD, STIMULUS etc)
- ☐ Other

47. Please list the members of your DBS Committee. Select all that apply.

- ☐ Movement Disorders Neurologist
- ☐ Neurosurgeon
- ☐ Neuropsychologist
- ☐ Psychiatrist / Neuropsychiatrist
- ☐ Registered Nurse
- ☐ Nurse Practitioners
- ☐ Physician Assistants
- ☐ Residents / Fellows
- ☐ PT, OT and SLP
- ☐ Social Workers
- ☐ Case Managers
- ☐ Financial Counselors

DBS Procedure

Please clarify the intra-operative DBS procedures at your center

48. What intra-operative technique(s) are utilized to evaluate and confirm micro-/macro- electrode position? Select all that apply. If no specific intra-operative technique is utilized, you may skip this question.

- [ ] Microelectrode Recording (MER)
- [ ] Image Guidance - CT
- [ ] Image Guidance - MRI

49. If MER is utilized, who performs the recording and analysis? Please select all that apply

- [ ] Neurologist
- [ ] Neurosurgeon
- [ ] Physiologist
- [ ] Other

50. Who performs the pre-operative and peri-operative stereotactic planning for the selected DBS target? Please select all that apply.

- [ ] Neurologist
- [ ] Neurosurgeon
- [ ] Physiologist
- [ ] Radiologist
- [ ] Representative from Planning Hardware / Software company
- [ ] Other

51. In treating Parkinson Disease, what approximate percentage of patients at your center are implanted at the following targets?

	0-20%	21-40%	41-60%	61-80%	81-100%	N/A
Subthalamic Nucleus (STN)	◯	◯	◯	◯	◯	◯
Globus pallidus internus (GPi)	◯	◯	◯	◯	◯	◯
Ventral intermediate thalamus (Vim)	◯	◯	◯	◯	◯	◯
Pedunculopontine nucleus (PPN)	◯	◯	◯	◯	◯	◯
Caudal zona incerta (cZi)	◯	◯	◯	◯	◯	◯
Thalamic centromedian parafasicular complex (CM-Pf)	◯	◯	◯	◯	◯	◯
Substantia Nigra Pars Reticulate (SNr)	◯	◯	◯	◯	◯	◯
Multiple Targets simultaneously	◯	◯	◯	◯	◯	◯

Post-Implantation and Follow-Up Care

Please clarify how your center deals with Post-Operative DBS Care and Follow-up

52. When is post-operative imaging obtained?

- ○ Within 24 hours
- ○ 1-3 days
- ○ 3-7 days
- ○ >7 days
- ○ Not routinely obtained (unless there are expected symptoms / signs)

53. What modality of post-operative imaging is used? (Please select all that apply. You may skip this question is you selected "Not routinely obtained ..." above)

- ☐ CT
- ☐ MRI
- ☐ Ventriculography

54. Who provides feedback to the referring physician regarding clinical efficacy?

- ○ We do not provide feedback to the referring physician
- ○ Movement Disorders Neurologist
- ○ Neurosurgeon

55. Is there routine post-operative evaluation for mood or cognitive disability or sequela?

- ○ Yes
- ○ No

56. How often are post-operative visits in the first year (i.e. for DBS programming, medication adjustment, mood / cognitive screening)? Multiple answers may be selected for initial post-operative period and later follow-up.

- [ ] Every 1 month
- [ ] Every 2 months
- [ ] Every 3 months
- [ ] Every 4 months
- [ ] Every 6 months
- [ ] A pre-specified schedule (e.g. at 1 month, 3 month, 6 month and 12 month post-DBS)
- [ ] No specific routine follow-up is required for DBS. DBS is checked whenever needed or during usual PD follow-up visit.

57. Do any of the following participate in routine post-operative evaluations after DBS implantation?

- [ ] PT, OT or SLP
- [ ] Social Workers
- [ ] Case Managers
- [ ] Psychiatrists / Neuropsychiatrists
- [ ] Neuropsychologists
- [ ] None of the Above

58. Does your Center possess a formal DBS specific Mortality-Morbidity Conference?

- ○ Yes
- ○ No

Comments and Suggestions

Please feel free to provide any additional comments or suggestions about your DBS referral process or this survey. Thank you very much for participating.

59. You, now, have the chance to revise your opinion regarding your choices in the survey. Please select one of the following:

- ◯ I have responded on behalf of our center / team / group practice and these choices reflect consensus among our team.
- ◯ I have responded as an individual, it is possible that colleagues in my group / team / center would respond differently.

60. Do you have any other comments, questions, or concerns?
