# Supplementary material for: Global Variability in Deep Brain Stimulation Practices for Parkinson’s Disease
Source: Front Hum Neurosci. 2021 Mar 31;15:667035. doi: 10.3389/fnhum.2021.667035 (PMC8044366; doi:10.3389/fnhum.2021.667035)
Supplement: Supplementary file 2 [file Data_Sheet_2.DOCX]

**Supplementary file 2**

Figure 1: Specialty/ training of respondents

Figure 2: Effect of mood and neurocognitive evaluation on “default” DBS target selection

Color represents frequency that mood or cognitive evaluations affect target selection while x-axis shows percentage of responding centers within each frequency bin. *Example: Neurocognitive evaluation affects target selection in <10% of cases at 24% of centers.*

Figure 3: Effect of mood and neurocognitive evaluation on staging of DBS procedures

Percentages in clock-wise orientation represent frequency of cases in which mood or neurocognitive evaluations affect staging of DBS leads at a given center. Rose plot pedal levels show percentage of centers in each frequency group. *Example: Approximately 20% of centers report that neurocognitive evaluation affects the decision to stage lead procedures 51-75% of the time.*
